# Serum Cortisol Is Associated With Cerebral Small Vessel Disease-Related Brain Changes and Cognitive Impairment

**DOI:** 10.3389/fnagi.2021.809684

**Published:** 2022-01-21

**Authors:** Qianwen Qiu, Xirui Zhou, Lingshan Wu, Yi Zhang, Zhiyuan Yu, Minghuan Wang, Hao Huang, Xiang Luo, Dengji Pan

**Affiliations:** Department of Neurology, Tongji Hospital, Tongji Medical College, Huazhong University of Science and Technology, Wuhan, China

**Keywords:** serum cortisol, cerebral small vessel disease, neuroimaging markers, cognitive function, total CSVD burden

## Abstract

**Objective:**

To evaluate the relationship between serum cortisol, cerebral small vessel disease (CSVD) neuroimaging markers, and cognitive performance.

**Methods:**

We recruited patients over 50 years old who attended our hospital for physical examination between November 2020 and July 2021. All participants were subject to brain magnetic resonance imaging (MRI), serum cortisol examination, and the Montreal cognitive function assessment (MoCA). On brain MRI, we scored the presence of each marker of CSVD, including white matter hyperintensity (WMH), lacunes, cerebral microbleeds (CMBs), and enlarged perivascular spaces (EPVS). One point was awarded for the presence of each marker, producing a score between 0 and 4.

**Results:**

In total, 158 participants were included in this study with a mean age of 60.5 (56.0–66.3) years; 55.1% were male. In the multivariable analyses, serum cortisol level was an independent predictor of WMH severity, the presence of lacunes/CMBs, moderate-severe EPVS and total CSVD burden after adjusting for confounding factors. Serum cortisol level had positive associations with periventricular/deep Fazekas score, burdens of lacunes/CMBs, moderate-severe EPVS, and total CSVD burden in dose-dependent manner, and was an independent predictor of cognitive impairment. Furthermore, the results of the receiver operating characteristic (ROC) curve analysis revealed an area under curve (AUC) of 0.745 with 64.1% sensitivity and 82.5% specificity, and an AUC of 0.705 with 52.1% sensitivity and 85.5 specificity of cortisol in detecting patients with high CSVD burden and MCI, respectively.

**Conclusions:**

Serum cortisol level is independently associated with each CSVD MRI markers, total CSVD burden and cognitive impairment. These findings provide clues for pathological mechanisms and suggest serum cortisol as a promising biomarker associated with CSVD.

## Introduction

Cerebral small vessel disease (CSVD) is a widespread cerebrovascular disease that shares common clinical manifestations, neuroimaging markers, and neuropathological findings thought to result from changes in the pial and parenchymal microcirculations (Pantoni, 2010). CSVD accounts for up to 25% of strokes and contributes to future risk of both stroke and dementia (Wardlaw et al., 2013a, 2019), seriously increasing the burden of CSVD on society. A recently published international consensus—STRIVE (STandards for ReportIng Vascular changes on nEuroimaging) —outlined the changes related to CSVD that may be reflected on an brain magnetic resonance imaging (MRI) scan: white matter hyperintensity (WMH), lacunes of presumed vascular origin, cerebral microbleeds (CMBs), and enlarged perivascular spaces (EPVS) (Wardlaw et al., 2013b). These neuroimaging surrogates are varied but frequently concurrent, suggesting a potential shared mechanism (Vilar-Bergua et al., 2016).

Cortisol is produced via activation of the hypothalamic-pituitary-adrenal axis in response to stress. The normal physiological cortisol secretion peaks in the morning and displays a nadir in the evening, a circadian cycle critical for healthy cognitive aging (McEwen and Gianaros, 2010; Prenderville et al., 2015). The lipophilicity of cortisol allows for easy access across the blood-brain barrier (BBB), and the long-term elevated cortisol negatively influences the brain structure and function of humans or animals (Prenderville et al., 2015; Jiang et al., 2017). For example, neuroimaging studies demonstrate that high cortisol in patients with Cushing's syndrome leads to decreased regional blood supply levels and neural activity, which is manifested as diffuse WMH on MRI (Pires et al., 2015; Zhang et al., 2021). Moreover, high cortisol levels were reported to have neurotoxic effects on the hippocampus and be related to atrophy, eventually aggravating cognitive impairment and dementia development in the elderly (Byers and Yaffe, 2011; Geerlings et al., 2015; Echouffo-Tcheugui et al., 2018).

Exposure to high levels of cortisol also increases the risk and deteriorates the prognosis of acute cardiovascular associated with cerebral hypoperfusion, endothelial dysfunction and atherosclerosis such as stroke, transient ischemic attack and coronary heart disease (Walker, 2007; Everson-Rose et al., 2014; Casas et al., 2017). At present, endothelial dysfunction, blood-brain barrier and amyloid deposition are widely considered to be important mechanisms leading to CSVD, which may result in the occurrence of CSVD together with genetic factors and chronic hypoperfusion (Wardlaw et al., 2013b). However, less research exists on the relationship between cortisol and CSVD lesions. Here, we aimed to comprehensively evaluate the relationship between serum cortisol levels and CSVD. We also compared serum cortisol levels among individuals with each component of CSVD based on their burden in an effort to explore the underlying pathological mechanism of CSVD.

## Methods

### Study Population

This study was based on the registration of inpatients for physical examinations in the Department of Neurology, Tongji Hospital, Tongji Medical College, Huazhong University of Science and Technology from November 2020 to July 2021. Subjects attended to the neurological clinic and were electively admitted to hospital for cognitive impairment, headache, dizziness or with the fear of stroke due to the presence of risk factors. They were screened according to our inclusion and exclusion criteria. The inclusion criteria were that patients over 50 years old agreed to receive serum cortisol test, participate in our study, and provided signed informed consent. Subjects were excluded according to the following criteria: (1) dementia; (2) intracranial hemorrhage; (3) intracranial space occupying lesion; (4) prominent visual or hearing impairment; (5) any MRI contraindications; (6) use of exogenous glucocorticoids; (7) extra- or intracranial large artery stenosis >50%; and (8) history of anxiety/depression or sleep disorder.

All participants received broad physical examinations, including MRI, magnetic resonance angiography (MRA), routine laboratory examinations, and neuropsychological assessment [Montreal Cognitive Assessment Test (MoCA)].

### Standard Protocol Approval, Registration, and Patient Consent

This study was approved by the Tongji Hospital Ethics Committee (No. 2019-S105). Written informed consent was obtained from all patients.

### Clinical Assessment

The baseline characteristics and clinical factors of all participants were recorded in detail, including sex, age, body mass index (BMI), education status, medical history, and history of current drinking or smoking. Hypertension was defined as having systolic blood pressure (SBP) ≥140 mm Hg, or diastolic blood pressure (DBP) ≥90 mm Hg, or taking antihypertensive medication. Laboratory tests including glycosylated hemoglobin A1c (HbA1c), lipid profile, and serum cortisol levels were also evaluated after 12 h overnight fasting. To measure serum cortisol levels, venous samples were collected in serum separation tubes and were separated with centrifugation. Next, serum cortisol levels were measured by a chemiluminescent microparticle immunoassay within 2 h of collection. Generally, the blood sample was collected between 6:00 and 7:30 am.

### Radiologic Assessment

MRI scans of all participants were acquired on a single 3.0 T scanner (Signa, GE Healthcare of America). We obtained the following images for each patient: T1-weighted, T2-weighted, T2 fluid-attenuated inversion recovery (FLAIR), diffusion weighted imaging sequences, susceptibility weighted imaging sequences, and 3-dimensional time-of-flight MRA. The parameters of conventional MRI sequences are shown in Supplementary Table 1. Available scans were rated by two radiologists trained in MRI assessments and blinded to the clinical data. Interrater reliability tests were performed in 30 subjects for each CSVD marker assessment, and the κ = 0.762–0.895 indicating good reliability. Any disagreements were resolved through a discussion with a third rater.

WMH were diagnosed and scored using Fazekas scale (0–6) (Fazekas et al., 1998): periventricular white matter hyperintensities (PVWMH, range 0–3) and deep white matter hyperintensities (DWMH, range 0–3). The overall WMH severity was identified as follows: none WMH (Fazekas score 0), mild WMH (Fazekas score 1–2), moderate WMH (Fazekas score 3–4), and severe WMH (Fazekas score 5–6) (Su et al., 2017). Lacunes of presumed vascular origin displayed as similar signal characteristics with cerebrospinal fluid on T1WI and T2WI sequences, with round or oval lesions of 3–15 mm in diameter. CMBs were defined as round or oval lesions ≤ 10 mm in diameter on SWI sequences (Wardlaw et al., 2013b). EPVS were defined as round, oval, or linear lesions, < 3 mm diameter, following the territory of a perforating arteriole, with signals similar to cerebral spinal fluid (CSF) (Wardlaw et al., 2013b). A validated 4-point visual scale (0 = none, 1 = 1–10 EPVS, 2 ≥ 20 EPVS, 3 = 40 EPVS, and 4 ≥ 40 EPVS) was used to rate EPVS at the center of semiovale (CS) and basal ganglia (BG). At both levels, we rated the unilateral slice containing the greatest number of EPVS, and defined moderate-severe EPVS as score ≥ 2 in BG (Charidimou et al., 2014). We used the recently developed scale to represent the total burden of CSVD by counting each neuroimaging feature (range 0–4) (Staals et al., 2014). One point was awarded for each of the following items: Fazekas score ≥ 2 in DWMH and/or Fazekas = 3 in PVWMH; ≥ 1 lacune; ≥ 1 CMBs; EPVS for grades 2–4 in BG. High CSVD burden was defined as total CSVD score ≥ 2 (Li et al., 2020).

### Neuropsychological Assessment

The Chinese version of MoCA (0–30 points) was used to evaluate the global cognitive function of participants. One additional point was added to MoCA scores for subjects with <12 schooling years to reduce the potential interference of education on our results. A cut off score of <21 or 26 was used for dementia or mild cognitive impairment, respectively. Hamilton Anxiety Scale 14 items (HAMA-14) and Hamilton Depression Scale 21 items (HAMD-21) were used to assess the appearance of anxiety and depression. The total score of HAMA or HAMD is operationally classified into severe anxiety or severe depression (score ≥ 14). Pittsburgh Sleep Quality Index (PSQI) was used to evaluate sleep disorders. Generally, a global score of >8 could be thought severe sleep problem. Each scale was jointly inspected and assessed by two staff who had received formal training. They scored individually, and consistency reached ≥90%.

### Statistical Analysis

Continuous variables with non-normal distributions are presented medians (interquartile range, IQR; the range between the 25th and 75th percentiles). Categorical variables are presented with case (percentage). Univariate comparisons were evaluated using Mann Whitney U test for continuous variables and chi-square test for categorical variables. All participants were dichotomized into lower and higher cortisol groups based on their median value of serum cortisol in an effort to compare characteristics between the two groups. Ordered logistic regression model or binary logistic logistic regression model (forward LR) was chosen to investigate whether serum cortisol level (as a continuous variable) was an independent risk factor for CSVD MRI markers and total CSVD burden. Demographic and confounding factors were adjusted in the model as follows: the model 1 was adjusted for age and sex; and the model 2 was adjusted for age, sex, BMI, SBP, DBP, current smoking, current drinking, TC, HbA1c, HAMA, HAMD. To assess the dose-dependence of the relationships between serum cortisol level and CSVD MRI markers, the Mann-Whitney U, Kruskal-Wallis, and Jonckheere-Terpstra test were used to compare mean serum cortisol levels among individuals with varying CSVD MRI markers. Simple linear regression analyses were used to identify factors (note: serum cortisol was used as a continuous variable) affecting MoCA scores. We then introduced the moderately significant variables (*p* < 0.05) in univariate analyses into multivariate analyses (stepwise forward). Multicollinearity among the independent variables were analyzed using the variance inflation factor. There was no multicollinearity between suspected predictor variables. The receiver operating characteristic (ROC) curve analysis was used to determine the area under curve (AUC) and cut-off values of serum cortisol. All statistical analyses in the study were performed using SPSS version 22 (IBM corporation, Armonk, NY). Values of *p* < 0.05 were considered statistically significant.

## Results

### Demographics and Clinical Characteristics

**Figure 1:**
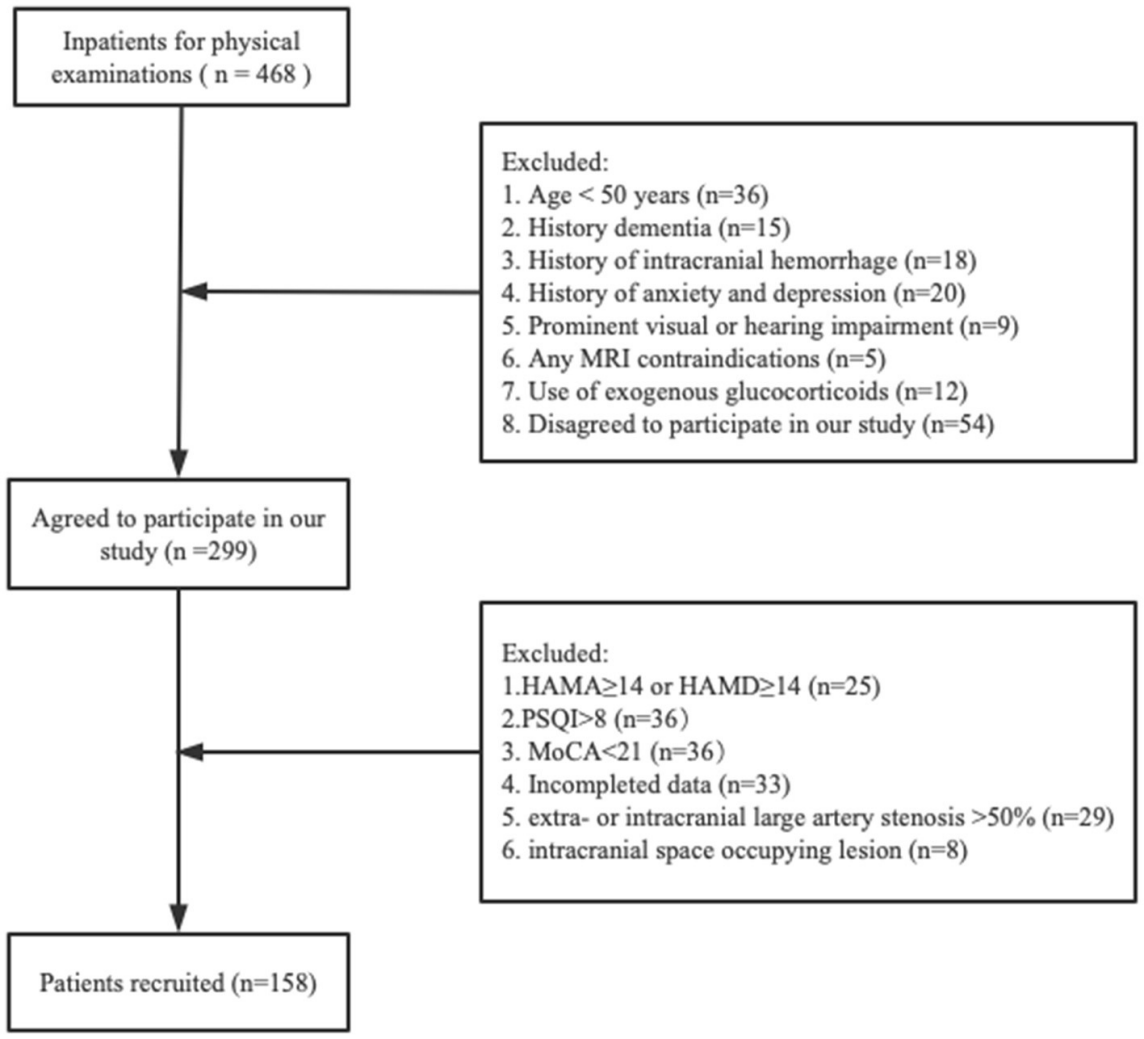
Flowchart of participants selection. HAMA, Hamilton Anxiety Scale; HAMD, Hamilton Depression Scale; PSQI, Pittsburgh Sleep Quality Index; MoCA, Montreal Cognitive Assessment.

A total of 468 inpatients were registered for physical examination, among whom 158 patients remained after applying our inclusion and exclusion criteria (Figure 1). Characteristics of all participants are shown (Table 1). The included subjects with a mean age of 60.5 (56.0–66.3) years; 55.1% were male. Imaging frequencies varied among the CSVD markers: none WMH [26 (16.5%)], mild WMH [43 (27.2%)], moderate WMH [39 (24.7%)], severe [50 (31.6%)], lacunes [72 (45.6%)], CMBs [57 (36.1%)], and moderate-severe EPVS [53 (33.5%)]. The median level of serum cortisol was 13.6 (10.3–16.3) ug/dL. Study participants were dichotomized into lower and higher cortisol groups. Notably, individuals in the higher serum cortisol group presented with higher frequencies of hypertension, moderate-severe WMH, lacunes, CMBs, moderate-severe EPVS and lower MoCA scores than the lower serum cortisol group; there was no statistically significant difference in other characteristics. The excluded groups had lower MoCA scores and higher HAMA scores than the included groups. No other significant differences were found between the two groups for clinical characteristics (Supplementary Table 2).

**Table 1:** Baseline characteristics of study population.

	**Total (*n* = 158)**	**Lower cortisol group < 13.6**	**Higher cortisol group ≥ 13.6**	***p*-Value**
		**(*n* = 79)**	**(*n* = 79)**	
Age, years, median (IQR)	60.5 (56.0–66.3)	60.0 (56.0–65.0)	61.0 (56.0–67.0)	0.412
Sex, male, n (%)	87 (55.1)	39 (49.4)	48 (60.8)	0.150
BMI, Kg/m^2^, median (IQR)	23.2 (21.5–25.5)	23.1 (21.7–25.8)	23.4 (21.3–25.0)	0.765
Hypertension, n (%)	82 (51.9)	34 (43.0)	48 (60.8)	0.026
SBP, mm Hg, median (IQR)	128.0 (121.0–138.0)	128.0 (122.0–136.0)	127.0 (118.0–140.0)	0.969
DBP, mm Hg, median (IQR)	75.0 (70.8–82.0)	75.0 (71.0–80.0)	77.0 (69.0–83.0)	0.594
Diabetes, n (%)	47 (29.7)	19 (24.1)	28 (35.4)	0.117
Hyperlipidemia, n (%)	88 (55.7)	44 (55.7)	44 (55.7)	1.000
Using of antihypertensive drugs, n (%)	72 (45.6)	31 (39.2)	41 (51.9)	0.110
Using of statin drugs, n (%)	66 (41.8)	34 (43.0)	32 (40.5)	0.747
Current smoking, n (%)	42(26.6)	16 (20.3)	26 (32.9)	0.072
Current drinking, n (%)	46 (29.1)	18 (22.8)	28 (35.4)	0.080
Education (high school or above), n (%)	56 (35.4)	27 (34.2)	29 (36.7)	0.739
TC, mmol/L, median (IQR)	3.6 (3.1–4.3)	3.6 (3.2–4.2)	3.6 (2.9–4.6)	0.727
TG, mmol/L, median (IQR)	1.2 (0.9–1.6)	1.2 (0.9–1.7)	1.1 (0.8–1.5)	0.222
HDL, mmol/L, median (IQR)	1.1 (0.9–1.2)	1.1 (0.9–1.2)	1.1(0.9–1.2)	0.821
LDL, mmol/L, median (IQR)	2.1 (1.7–2.8)	2.1 (1.7–2.7)	2.1 (1.7–2.9)	0.906
HAb1c, %, median (IQR)	5.8 (5.6–6.2)	5.9 (5.6–6.1)	5.8 (5.6–6.4)	0.264
Serum cortisol, ug/dL, median (IQR)	13.6 (10.3–16.3)	10.4 (8.2–11.6)	16.3 (14.3–17.9)	<0.001
Fazekas grade				
None (0), n (%)	26 (16.5)	20 (25.3)	6 (7.6)	<0.001
Mild (1–2), n (%)	43 (27.2)	29 (36.7)	14 (17.7)	
Moderate (3–4), n (%)	39 (24.7)	13 (16.5)	26 (32.9)	
Severe (5–6), n (%)	50 (31.6)	17 (21.5)	33 (41.8)	
Lacunes, n (%)	72 (45.6)	24 (30.4)	48 (60.8)	<0.001
CMBs, n (%)	57 (36.1)	14 (17.7)	43 (54.4)	<0.001
Moderate-severe EPVS, n (%)	53 (33.5)	14 (17.7)	39 (49.4)	<0.001
Total CSVD burden				
0, n (%)	47 (29.7)	37 (46.8)	10 (12.6)	<0.001
1, n (%)	33 (20.3)	18 (22.7)	15 (18.9)	
2, n (%)	26 (16.4)	12 (15.1)	14 (17.7)	
3, n (%)	27 (17.0)	8 (10.1)	19 (24.0)	
4, n (%)	25 (15.8)	4 (5.0)	21 (26.5)	
HAMA, score, median (IQR)	7.0 (5.0–8.0)	8.0 (5.0–8.0)	7.0 (5.0–8.0)	0.160
HAMD, score, median (IQR)	7.0 (6.0–8.0)	7.0 (5.0–8.0)	7.0 (6.0–8.0)	0.909
MoCA, score, median (IQR)	25.0 (23.0–26.0)	26.0 (24.0–27.0)	24.0 (22.0–25.0)	<0.001

*BMI, body mass index; SBP, systolic blood pressure; DBP, diastolic blood pressure; TC, total cholesterol; TG, triglyceride; HDL, high-density lipoprotein; LDL, low-density lipoprotein; HAb1c, hemoglobin A1c; WMH, white matter hyperintensity; CMBs, cerebral microbleeds; EPVS, enlarged perivascular spaces; HAMA, Hamilton Anxiety Scale; HAMD, Hamilton Depression Scale; MoCA, Montreal Cognitive Assessment*.

### Serum Cortisol and CSVD MRI Markers

The results of the logistic regression analyses are shown in Table 2. In the model 1, serum cortisol was an independent predictor of WMH severity (adjusted odds ratio [aOR] = 1.169; 95% confidence interval [CI] = 1.085–1.260, *p* = 0.001), lacunes (aOR = 1.211; 95% CI = 1.102–1.330, *p* < 0.001), CMBs (aOR = 1.224; 95% CI = 1.110–1.351, *p* < 0.001), moderate-severe EPVS (aOR = 1.306; 95% CI = 1.168–1.459, *p* < 0.001) and total CSVD burden (aOR = 1.274; 95% CI = .176–1.379, *p* < 0.001) after adjustment for confounding factors. In the model 2, serum cortisol was an independent predictor of WMH severity (aOR = 1.221; 95% CI = 1.123–1.328, *p* < 0.001), lacunes (aOR = 1.219; 95% CI = 1.106–1.343, *p* < 0.001), CMBs (aOR = 1.235; 95% CI = 1.118–1.365, *p* < 0.001), moderate-severe EPVS (aOR = 1.306; 95% CI = 1.17–1.457, *p* < 0.001) and total CSVD burden (aOR = 1.288; 95% CI = 1.183–1.401, *p* < 0.001) after adjustment for confounding factors.

**Table 2:** Multivariate logistic regression analyses between serum cortisol levels and CSVD MRI markers.

	**Model 1**		**Model 2**	
	**OR (95% CI)**	***p-*value**	**OR (95% CI)**	***p-*value**
Fazekas grade^a^	1.169 (1.085–1.260)	<0.001	1.221 (1.123–1.328)	<0.001
Lacunes^b^	1.211 (1.102–1.330)	<0.001	1.219 (1.106–1.343)	<0.001
CMBs^b^	1.224 (1.110–1.351)	<0.001	1.235 (1.118–1.365)	<0.001
Moderate-severe EPVS^b^	1.306 (1.168–1.459)	<0.001	1.306 (1.171–1.457)	<0.001
CSVD burden^a^	1.274 (1.176–1.379)	<0.001	1.288 (1.183–1.401)	<0.001

*Model 1: adjusted for age and sex*.

*Model 2: adjusted for age, sex, BMI, SBP, DBP, current smoking, current drinking, TC, HbA1c, HAMA, HAMD*.

a *Ordinal logistic regression*.

b *Binary logistic regression*.

*Model 1 (adjusted for age and sex) and Model 2 (age, sex, BMI, SBP, DBP, current smoking, current drinking, TC, HbA1c, HAMA, HAMD)*.

**Figure 2:**
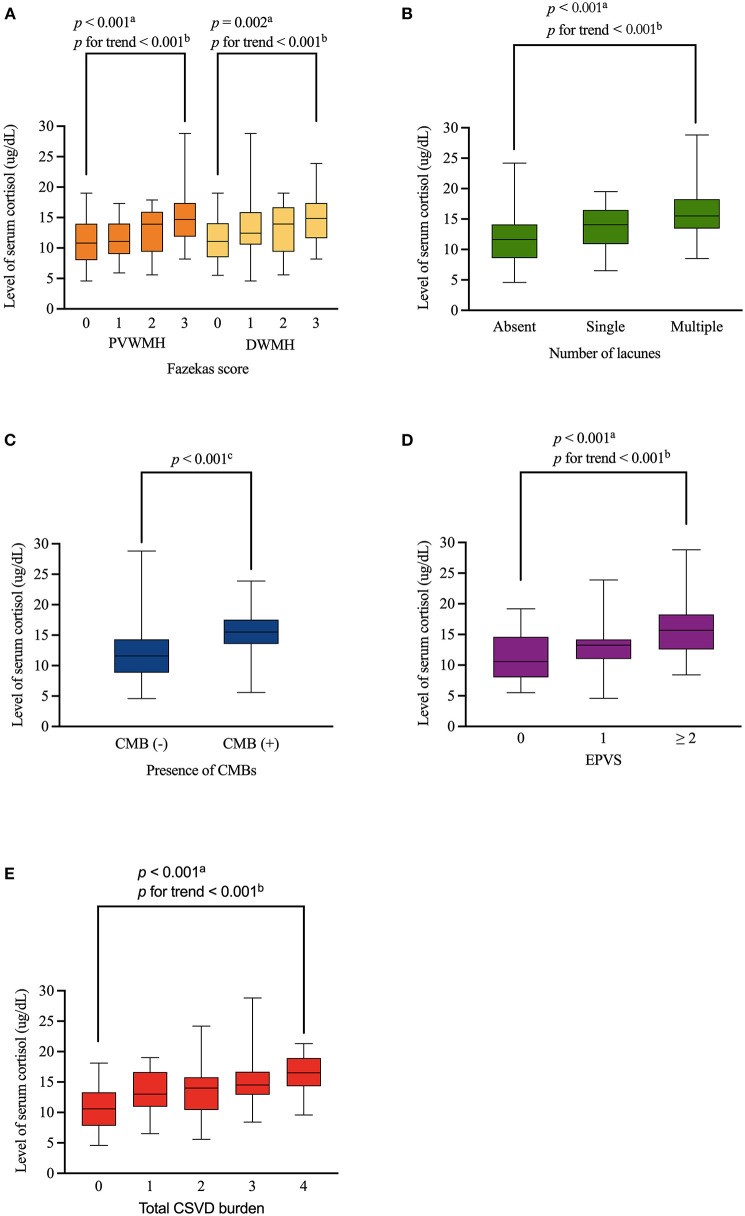
Distribution of levels of serum cortisol according to WMH, lacunes, CMBs, EPVS and total CSVD burden. Serum cortisol level was positively associated with Fazekas score (both of PVWMH and DWMH), numbers of lacunes, presence of CMBs, moderate-severe EPVS, and total CSVD burden in a dose-dependent manner (**A–E**, respectively). Boxplot elements: center line represents the median, box bounds represent the 25th and 75th percentile, and whiskers represent minimum and maximum respectively. PVWMH, periventricular white matter hyperintensity; DWMH, deep white matter hyperintensity; CMBs, cerebral microbleeds; EPVS, enlarged perivascular spaces. ^a^Kruskal-Wallis test. ^b^Jonckheere-Terpstra test. ^c^Mann-Whitney U test.

An assessment of the relationship between serum cortisol levels and the burdens of different MRI markers of CSVD showed a positive dose-dependent correlation between serum cortisol levels and the Fazekas score in both of PVWMH (*p* < 0.001, *p* for trend < 0.001) and DWMH (*p* = 0.002, *p* for trend < 0.001). Serum cortisol level displayed a close relationship with numbers of lacunes (*p* < 0.001, *p* for trend < 0.001), presence of CMBs (*p* < 0.001), moderate-severe EPVS (*p* < 0.001, *p* for trend < 0.001), and total CSVD burden (*p* < 0.001, *p* for trend < 0.001) (Figure 2).

### Serum Cortisol and Cognitive Outcomes

**Figure 3:**
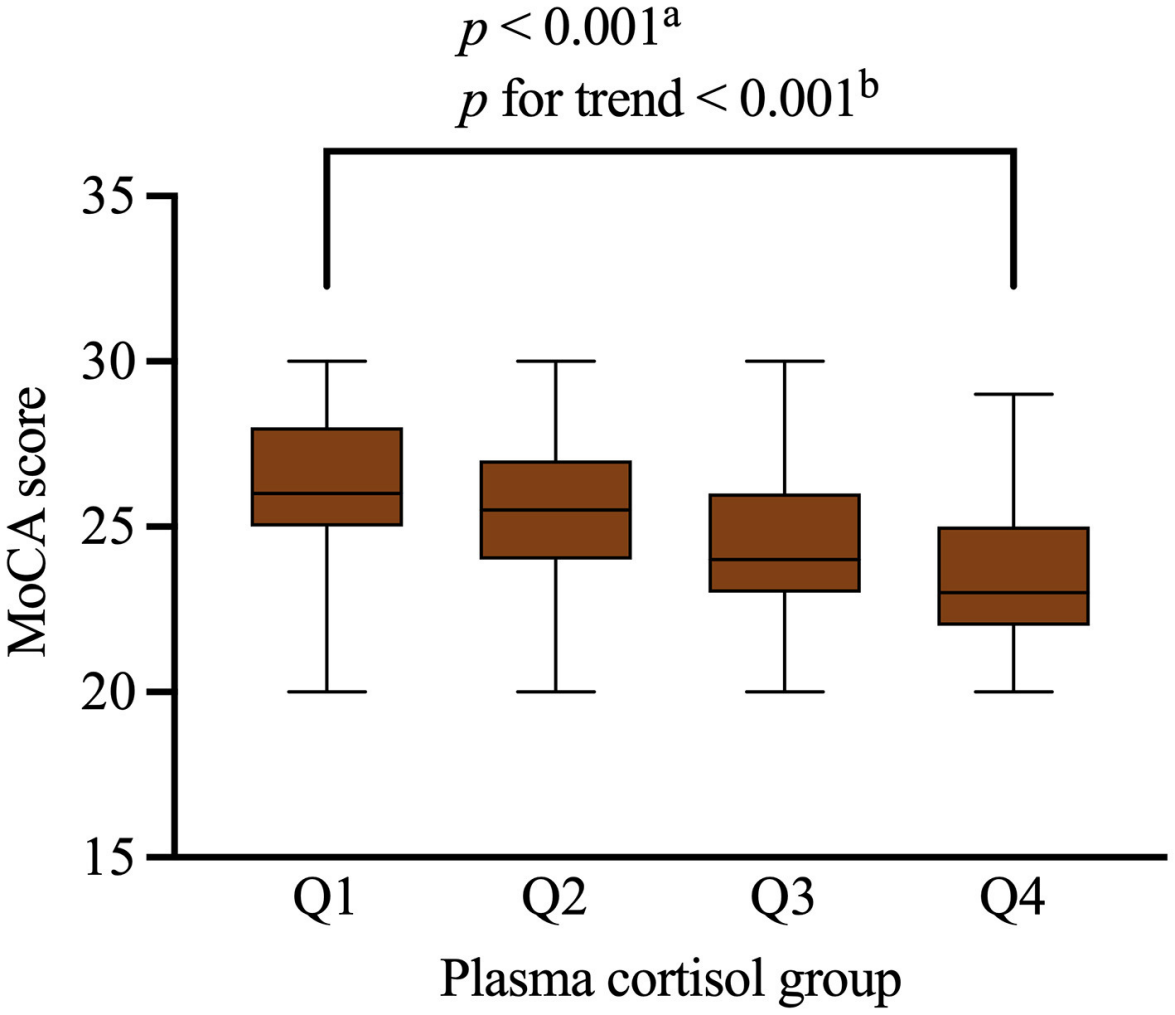
MoCA scores of the participants with different quartiles of serum cortisol levels. Serum cortisol levels were closely related to cognitive impairment in the participants. Serum cortisol quartiles in the subjects were: Q1 < 10.3 ug/dL, Q2 = 10.3–13.6 ug/dL, Q3 = 13.6–15.0 ug/dL, Q4 ≥ 15.0 ug/dL. Boxplot elements: center line represents the median, box bounds represent the 25th and 75th percentile, and whiskers represent minimum and maximum respectively. MoCA, Montreal Cognitive Assessment. ^a^Kruskal-Wallis test. ^b^Jonckheere-Terpstra test.

Table 3 shows the results of the linear regression analyses of the associations between possible factors and MoCA scores, indicating that serum cortisol remained an independent predictor of cognitive impairment of participants (β = −0.178, 95% CI= −0.261– −0.094, *p* < 0.001). We stratified the levels of serum cortisol into quartiles. Notably, increasing serum cortisol levels displayed a closed relationship with cognitive function of patients in a significant downward trend (*p* < 0.001, *p* for trend *p* < 0.001) (Figure 3).

**Table 3:** Multiple linear regression analyses between serum cortisol levels and MoCA scores.

	**Univariate analysis**		**Multivariate analysis^a^**	
	**β (95% CI)**	***p*-value**	**β (95% CI)**	***p*-value**
Age	−0.068 (−0.118–−0.017)	0.009	−0.053 (−0.098–−0.008)	0.021
Sex	−0.298 (−1.078–0.483)	0.452	–	–
Education	1.644 (0.874–2.414)	<0.001	1.728 (1.038–2.417)	<0.001
BMI	−0.094 (−0.231–0.044)	0.180	–	–
Hypertension	−0.279 (−1.056–0.498)	0.479	–	–
Diabetes	−0.016 (−0.867–0.834)	0.970	–	–
Hyperlipidemia	−0.360 (−1.141–0.421)	0.364	–	–
Current smoking	0.092 (−0.788–0.971)	0.837	–	–
Current drinking	0.066 (−0.790–0.921)	0.880	–	–
HAMA	0.086 (−0.117–0.289	0.403	–	–
HAMD	0.001 (−0.212–0.214)	0.994	–	–
CSVD burden	−0.567 (−0.820–−0.315)	<0.001	−0.322 (−0.591–−0.032)	0.020
Serum cortisol	−0.215 (−0.303–−0.128)	<0.001	−0.154 (−0.246–−0.062)	0.001

*BMI, body mass index; HAMA, Hamilton Anxiety Scale, HAMD, Hamilton Depression Scale, MoCA, Montreal Cognitive Assessment*.

a *Adjusted with p < 0.05 in the univariate analysis (age, education, CSVD burden, and serum cortisol)*.

### ROC Curve Analysis of Serum Cortisol Predicting High CSVD Burden or MCI

**Figure 4:**
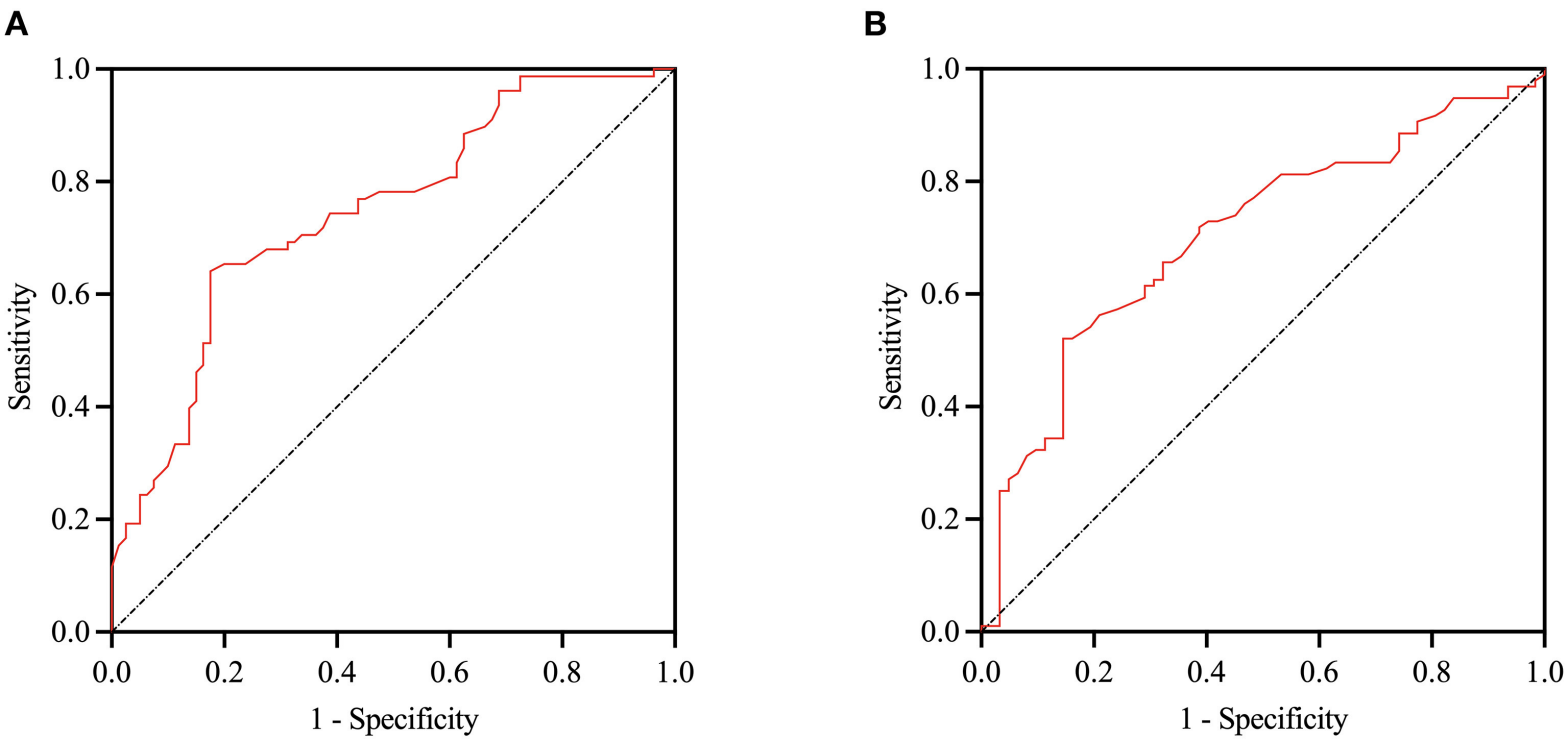
ROC curve of serum cortisol in identification of the patients with high CSVD burden and MCI. The AUC using cortisol to detect patients with high CSVD burden was 0.745 (95% CI 0.669–0.822, *p* < 0.001), and patients with MCI was 0.705 (95% CI, 0.623–0.787 *p* < 0.001) (**A,B** respectively). ROC, Receiver operating characteristicl; AUC, area under curve; MCI, mild cognitive impairment.

The ROC curve analysis indicated the potential diagnostic values of serum cortisol. We divided the subjects into high CSVD burden groups (defined as total CSVD score ≥ 2) and low CSVD groups. The AUC using serum cortisol to detect patients with high CSVD burden was 0.745 (95% CI, 0.669–0.822, *p* < 0.001). At a cut-off point of 14.15 μg/dL for serum cortisol, the sensitivity and specificity were 64.1% and 82.5% for predicting patients with high CSVD burden. In addition, we divided the subjects into MCI groups (defined as MoCA score <26) and healthy groups. The AUC using serum cortisol to detect patients with MCI was 0.705 (95% CI, 0.623–0.787, *p* < 0.001). At a cut-off point of 14.35 μg/dL for serum cortisol, the sensitivity and specificity were 52.1% and 85.5% for predicting patients with MCI (Figure 4).

## Discussion

In this study, we investigated the association between serum cortisol levels and CSVD for the first time in patients who underwent physical examinations in our hospital. Cortisol was reported to be associated with anxiety, depression and sleep quality, thus we used the corresponding scale to exclude participants with severe disorders respectively to reduce the interference with the results. Positive associations were consistent in the analyses of individual MRI markers of CSVD, total CSVD burden and cognitive impairment, indicating that elevated serum cortisol may lead to CSVD-related brain damage and then impaired cognitive function. Furthermore, ROC curve analysis showed that serum cortisol might be a promising biomarker associated with CSVD (AUC >0.7).

Consistent with previous studies, we found that higher level of cortisol was associated with greater WMH (Cox et al., 2015a; Pires et al., 2015). Moreover, increasing evidence also suggests that higher serum cortical levels contribute to endothelial dysfunction (García-Bueno et al., 2008; Duckles and Miller, 2010). Indeed, cortisol may impair endothelial function through the downregulation of endothelial nitric oxide synthase (eNOS) expression, eNOS deactivation, impeded nitric oxide (NO) actions, and enhanced NO degradation, together with vasoconstriction counteracting against NO-induced vasodilatation (Toda and Nakanishi-Toda, 2011). The potential outcome of this series of processes is the impairment of endothelium-dependent cerebral blood flow regulation (i.e., chronic hypoperfusion) (Hassan et al., 2003). eNO also prevents arteriosclerosis by inhibiting fibrosis and proliferation of vascular smooth muscle cells (Walker, 2007; Duckles and Miller, 2010). Therefore, chronic hypoperfusion state and arteriosclerosis induced by inhibition of eNO may result in WMH or lacunes.

Another explanation for our observation may involve eNO inhibition leading to BBB dysfunction. Disrupted BBB may result in leakage of fluid, plasma components and cells into neural tissues or the blockage of interstitial fluid clearance via the glymphatic pathway, and eventually lead to perivascular inflammation, demyelination and gliosis (Shi and Wardlaw, 2016; Wong et al., 2019). Thus, these events may result in the development of WMH, CMBs or EPVS. In addition, an elevated serum cortisol level may also lead to the accumulation of amyloid proteins. Generally, the presence of CMBs is associated with increased deposition or decreased clearance of β-amyloid (Aβ) protein via the glymphatic pathway (Pantoni, 2010; Wardlaw et al., 2013b). Recent findings revealed that high cortisol levels have also been associated with increased Aβ peptide in Alzheimer's disease (Ouanes and Popp, 2019), although these findings may be due to selection bias of the study populations. Interestingly, similar with the above findings, we observed a close relationship between higher serum cortisol level and the presence of CMBs. Finally, although the pathogenesis of CSVD is not completely clear, it is fundamental to keep in mind that all the features of CSVD are strictly inter-related. Proceeding from this point, we used the total CSVD score to assess the MRI burden of CSVD, which is clinically easy to use and might better capture the total subcortical microstructural brain damage from CSVD. Our findings underline the importance of a higher serum cortisol level as a risk factor for increased MRI burden of CSVD. Prior studies revealed that high serum cortisol level is related to hypertension, hyperlipidemia, hyperglycemia and other cerebrovascular risk factors, which are also risk factors for the development of CSVD. Therefore, our observations might be a coincidence caused by high serum cortisol levels and common risk factors in patients with CSVD.

We found that higher cortisol levels are associated with worse cognitive function. To date, WMH is considered to be an independent predictor of cognitive decline (Østergaard et al., 2016; Jokinen et al., 2020). One possible mechanism plays a role in the relationship between high levels of cortisol, CSVD and cognitive impairment: the propensity for cognitive impairment might have been mediated by the higher MRI burden of CSVD associated with the higher serum cortisol level. Therefore, we introduced CSVD burden as covariates in multivariate analyses, and found cortisol levels remained independently associated with cognitive impairment. Moreover, high cortisol levels for long periods of time have been reported to be associated with lower brain volumes (particularly the hippocampus and prefrontal cortex), and have neurotoxic effects, which play an important role in cognitive impairment (Cox et al., 2015b; Echouffo-Tcheugui et al., 2018).

While we aimed to investigate the effects of elevated serum cortisol on CSVD, there were several limitations. First, this is a single-center observational study with limited samples. Thus, selection bias may have been present and similar studies should utilize larger samples. Second, because of constraints of cross-sectional analysis, we were unable to provide evidence for causality, and further prospective longitudinal evaluation is required. Finally, because we did not exclude healthy subjects who had fewer vascular or neurologic problems, it may have prohibited the evaluation of the relationship between high serum cortisol levels and CSVD components. Future studies with enlarged sample size and in particular more patients with CSVD are warranted.

## Conclusion

In summary, our study suggests that an elevated serum cortisol level is independently associated with each CSVD MRI markers, total CSVD burden and cognitive impairment. Although our findings should be validated in prospective studies with larger sample sizes, provide clues for pathological mechanisms and suggest serum cortisol as a promising biomarker associated with CSVD.

## Data Availability Statement

The original contributions presented in the study are included in the article/Supplementary Material, further inquiries can be directed to the corresponding author.

## Ethics Statement

This study was approved by the Tongji Hospital Ethics Committee (No. 2019-S105). The patients/participants provided their written informed consent to participate in this study.

## Author Contributions

QQ: drafting/revision of the manuscript for content, major role in the acquisition of data, study concept or design, and analysis or interpretation of data. XZ: major role in the acquisition of data and analysis or interpretation of data. LW, YZ, and ZY: major role in the acquisition of data and study concept or design. MW, HH, XL, and DP: study concept or design. All authors contributed to the article and approved the submitted version.

## Funding

This study was supported by the National Nature Science Foundation of China (81771341 to XL), Key Research and Development Program of Hubei Province (2020BCA070 to XL), the Application Foundation Frontier Special Project of Wuhan Science and Technology Bureau (2020020601012226 to XL), and the Flagship Program of Tongji Hospital (2019CR106 to XL).

## Conflict of Interest

The authors declare that the research was conducted in the absence of any commercial or financial relationships that could be construed as a potential conflict of interest.

## Publisher's Note

All claims expressed in this article are solely those of the authors and do not necessarily represent those of their affiliated organizations, or those of the publisher, the editors and the reviewers. Any product that may be evaluated in this article, or claim that may be made by its manufacturer, is not guaranteed or endorsed by the publisher.
