# Supplementary material for: Serum Cortisol Is Associated With Cerebral Small Vessel Disease-Related Brain Changes and Cognitive Impairment
Source: Front Aging Neurosci. 2022 Jan 21;13:809684. doi: 10.3389/fnagi.2021.809684 (PMC8814515; doi:10.3389/fnagi.2021.809684)
Supplement: Supplementary file 1 [file Table_1.DOCX]

**Table S1. Parameters for conventional MRI sequences.**

| **Sequences** | **T1WI** | **T2WI** | **FLAIR** | **DWI** | **SWI** |
| --- | --- | --- | --- | --- | --- |
| **TR, ms** | 2390 | 5200 | 7902 | 5400 | 30 |
| **TE, ms** | 10.69 | 107.41 | 140.45 | 90.30 | 20 |
| **FA, °** | 111 | 142 | 150 | 90 | 15 |
| **Slice thickness, mm** | 5 | 5 | 5 | 5 | 2 |
| **FOV, mm^2^** | 240 × 230 | 240 × 230 | 240× 230 | 230 × 230 | 230×230 |

Abbreviations: T1WI = T1-weighted imaging; T2WI = T2-weighted imaging; FLAIR = fluid-attenuated inversion recovery; DWI = diffusion-weighted imaging; TR = repetition time; TE = echo time; FA = flip angle; FOV = field of view.

**Table S2. Clinical characteristics of individuals included and excluded in the study.**

|  | **Included individuals**  **( n= 158)** |  | **Excluded individuals**  **(n = 141)** | ***p* value** |
| --- | --- | --- | --- | --- |
| **Age, years, median (IQR)** | 60.5 (56.0–66.3) |  | 63.0 (56.0–69.0) | 0.389 |
| **Sex, male, n (%)** | 87 (55.1) |  | 75 (53.2) | 0.746 |
| **BMI, Kg/m^2^ ,median (IQR)** | 23.2 (21.5–25.5) |  | 22.6 (21.3–24.6) | 0.129 |
| **Hypertension, n (%)** | 82 (51.9) |  | 82 (58.2) | 0.278 |
| **SBP, mm Hg, median (IQR)** | 128.0 (121.0–140.0) |  | 132.0 (122.0–141.0) | 0.118 |
| **DBP, mm Hg, median (IQR)** | 75.0 (70.0–82.0) |  | 76.0 (69.0–82.0) | 0.949 |
| **Diabetes, n (%)** | 47 (29.7) |  | 42 (29.8) | 0.994 |
| **Hyperlipidemia, n (%)** | 88 (55.7) |  | 79 (56.0) | 0.954 |
| **Using of antihypertensive**  **drugs, n (%)** | 72 (45.6) |  | 63 (44.7) | 0.877 |
| **Using of statin drugs, n(%)** | 66 (41.8) |  | 58 (41.1) | 0.911 |
| **Current smoking, n (%)** | 42 (26.6) |  | 34 (24.1) | 0.625 |
| **Current drinking, n (%)** | 46 (29.1) |  | 32 (22.7) | 0.207 |
| **Education (high school or above), n (%)** | 56 (35.4) |  | 38 (27.0) | 0.114 |
| **TC, mmol/L, median (IQR)** | 3.6 (3.1–4.3) |  | 3.8 (3.3–4.5) | 0.150 |
| **TG, mmol/L, median (IQR)** | 1.2 (0.9–1.6) |  | 1.2 (0.9–1.6) | 0.235 |
| **HDL, mmol/L, median (IQR)** | 1.1 (0.9–1.2) |  | 1.1 (0.9–1.3) | 0.622 |
| **LDL, mmol/L,median (IQR)** | 2.1 (1.7–2.8) |  | 2.3 (1.7–2.7) | 0.613 |
| **HAb1c, %, median (IQR)** | 5.8 (5.6–6.2) |  | 5.9 (5.7–6.2) | 0.100 |
| **Serum cortisol, ug/ dL,**  **median (IQR)** | 13.6 (10.3–16.3) |  | 13.9 (11.8–16.1) | 0.074 |
| **HAMA, score, median (IQR)** | 7.0 (5.0–8.0) |  | 8.0 (6.0–9.0) | 0.009 |
| **HAMD, score, median (IQR)** | 7.0 (6.0–8.0) |  | 7.0 (6.0–9.0） | 0.190 |
| **MoCA, score, median (IQR)** | 25.0 (23.0–26.0) |  | 24.0 (20.0–26.0) | < 0.001 |

Abbreviations: BMI = body mass index; SBP = systolic blood pressure; DBP = diastolic blood pressure; TC = total cholesterol; TG = triglyceride; HDL = high-density lipoprotein; LDL = low-density lipoprotein; HAb1c = hemoglobin A1c; HAMA = Hamilton Anxiety Scale; HAMD = Hamilton Depression Scale; MoCA = Montreal Cognitive Assessment.
